# Renal and Neurologic Benefit of Levosimendan *vs* Dobutamine in Patients With Low Cardiac Output Syndrome After Cardiac Surgery: Clinical Trial FIM-BGC-2014-01

**DOI:** 10.3389/fphar.2020.01331

**Published:** 2020-08-26

**Authors:** Jose L. Guerrero-Orriach, Alfredo Malo-Manso, Marta Ramirez-Aliaga, Ana Isabel Florez Vela, Manuel Galán-Ortega, Isabel Moreno-Cortes, Inmaculada Gonzalez-Almendros, Alicia Ramirez-Fernandez, Daniel Ariza-Villanueva, Juan Jose Escalona-Belmonte, Guillermo Quesada-Muñoz, Enrique Sepúlveda-Haro, Salvador Romero-Molina, Inmaculada Bellido-Estevez, Aurelio Gomez-Luque, Manuel Rubio-Navarro, Juan Alcaide-Torres, Concepcion Santiago-Fernandez, Lourdes Garrido-Sanchez, Jose Cruz-Mañas

**Affiliations:** ^1^ Institute of Biomedical Research in Malaga [IBIMA], Malaga, Spain; ^2^ Department of Anaesthesiology, Virgen de la Victoria University Hospital, Malaga, Spain; ^3^ Department of Pharmacology and Pediatrics, School of Medicine, University of Malaga, Malaga, Spain; ^4^ Unidad de Gestión Clínica de Endocrinología y Nutrición, Virgen de la Victoria University Hospital, Málaga, Spain; ^5^ CIBER Fisiopatología de la Obesidad y Nutrición-CIBEROBN, Instituto de Salud Carlos III, Málaga, Spain

**Keywords:** levosimendan, dobutamine, low cardiac output, kidney, cardiac surgery

## Abstract

**Background:**

Low-cardiac output syndrome (LCOS) after cardiac surgery secondary to systemic hypoperfusion is associated with a higher incidence of renal and neurological damage. A range of effective therapies are available for LCOS. The beneficial systemic effects of levosimendan persist even after cardiac output is restored, which suggests an independent cardioprotective effect.

**Methods:**

A double-blind clinical trial was conducted in patients with a confirmed diagnosis of LCOS randomized into two treatment groups (levosimendan *vs.* dobutamine). Monitoring of hemodynamic (cardiac index, systolic volume index, heart rate, mean arterial pressure, central venous pressure, central venous saturation); biochemical (e.g. creatinine, S100B protein, NT-proBNP, troponin I); and renal parameters was performed using acute kidney injury scale (AKI scale) and renal and brain ultrasound measurements [vascular resistance index (VRI)] at diagnosis and during the first 48 h.

**Results:**

Significant differences were observed between groups in terms of cardiac index, systolic volume index, NT-proBNP, and kidney injury stage at diagnosis. In the levosimendan group, there were significant variations in AKI stage after 24 and 48 h. No significant differences were observed in the other parameters studied.

**Conclusion:**

Levosimendan showed a beneficial effect on renal function in LCOS patients after cardiac surgery that was independent from cardiac output and vascular tone. This effect is probably achieved by pharmacological postconditioning.

**Clinical Trial Registration:**

EUDRA CT, identifier 2014-001461-27. https://www.clinicaltrialsregister.eu/ctr-search/search?query=2014-001461-27

## Highlights

Question: Do levosimendan and dobutamine for the treatment of LCOS have different neurological and renal effects? Finding: Significant variations were observed in the levosimendan group in relation to AKI stage after 24 and 48 h but not in cardiac index or renal blood flow.

Meaning: The beneficial effects of Levosimendan could be mediated by a postconditioning effect.

## Introduction

The incidence of low cardiac output syndrome (LCOS) after cardiac surgery is 10–20%. The effects of this syndrome on cardiac function are induced by splanchnic hypoperfusion, which requires treatment with inotropic drugs to restore organ perfusion (Lomivorotov et al., 2017).

LCOS is treated with inotropics, which improve cardiovascular function after volume optimization. Thus, inotropics restore cardiac function and an adequate blood flow in central and peripheral organs. The selection of the inotropic agent for the treatment of LCOS is based on the hemodynamic status of the patient. Examples of inotropic agents include beta-agonists and calcium sensitizers such as levosimendan (Bastien and Vallet, 2005; Mebazaa et al., 2010; Pérez Vela et al., 2012).

Cardiovascular response to levosimendan *vs* beta-agonists (such as dobutamine) in the treatment of LCOS has been assessed in a variety of studies (Follath et al., 2002; Alvarez et al., 2013; Gandham et al., 2013; Xuan et al., 2013). The scientific literature demonstrates that levosimendan improves renal function, increases survival rates, and causes fewer side effects (Yilmaz et al., 2007; Bragadottir et al., 2013; Yilmaz et al., 2013; Baysal et al., 2014; CHEETAH Study Group, 2018; Guerrero Orriach et al., 2019). Some studies suggest that the organ-protective effects of levosimendan are mediated by its action on potassium channels and mitochondria (Kopustinskiene et al., 2004; Du Toit et al., 2008; Soeding et al., 2011). In contrast, other studies point at its beneficial effects on organ perfusion as the underlying mechanism of its organ protective action (Sonntag et al., 2004; Metzsch et al., 2007).

The potential pre- and postconditioning properties of levosimendan have been explored in different studies (Du Toit et al., 2008; Stroethoff et al., 2019). LCOS is a clinical model of organ hypoperfusion, and restoration of organ perfusion might mimic ischemia-reperfusion injury. In this context, the postconditioning effect of the drugs administered for LCOS can be observed (Kübler and Haass, 1996; Buchholz et al., 2014; Heusch, 2015; Guerrero Orriach et al., 2018; Guerrero Orriach and Ramirez-Fernandez, 2019).

The objective of this study was to evaluate the systemic, neurological, and renal effects of levosimendan in patients with LCOS, as compared to dobutamine. The beneficial effects and mechanism of action of levosimendan on organs were assessed based on hemodynamic, biochemical, and ultrasound parameters.

## Materials and Methods

A double-blind clinical trial was conducted at *Hospital Universitario Virgen de la Victoria, Malaga, Spain.* This study was approved by the Institutional Review Board (CEI Malaga Norte). Written informed consent was obtained from all subjects participating in the trial. Prior to patient enrolment, the trial was registered with the European database of clinical trials EUDRACT under number 2014-001461-27 (Principal investigator: Jose Luis Guerrero Orriach, Date of registration: 13/01/2015). After informed consent was obtained and inclusion and exclusion criteria were applied, 60 patients were recruited and randomized at a 1:1 ratio into two treatment groups composed of 30 patients each. The clinical trial was monitored by the Instituto Biomédico de Málaga (IBIMA) and was conducted in accordance with GCP and European GDPR. This manuscript adheres to the applicable CONSORT guidelines.

Inclusion criteria were:

- Patients eligible for inotropic support for LCOS (cardiac index <2 L/min/m^2^; and/or SvO_2_ <65%), after optimization of volume.

Exclusion criteria:

- History of adverse reactions to levosimendan.
- Combined surgery (e.g. heart valve repair, carotid surgery).
- Hemodynamic instability.
- Preoperative renal impairment, estimated from the preoperative glomerular filtration rate (creatinine clearance <50 ml/min).
- Hypersensitivity to levosimendan or any of its excipients.
- Significant mechanical obstructions that may affect ventricular input and/or output.
- History of *torsade de pointes*.
- Cardiogenic shock (lactate >4 mmol/l).

Treatment 1: Levosimendan was administered by continuous infusion over 24 h until a dose of 12.5 mg was reached. Infusion rate: 0.1 mcg/kg/min.

Treatment 2: Dobutamine was administered at a starting infusion rate of 5 mcg/kg/min until hemodynamic targets were reached; then, the perfusion rate was increased by 2 mcg/kg/min, if necessary.

When diuresis was <1 ml/min, hemodynamics were optimized following this protocol: LCOS therapy was started, MAP values were maintained below 15% of basal values. If at the beginning of the treatment diuresis was <1 ml/kg/h for an hour, a 500 ml dose of Ringer lactate was administered for 30 min; if the outcome was not satisfactory, the process was repeated. If there was no response, a 10 mg dose of furosemide was administered every 30 min until a favorable response was achieved. When diuresis was >1 ml/kg/h, no diuretic drug was administered (Pérez Vela et al., 2012).

Five-lead ECG monitoring was performed in all patients; leads II and V were continuously recorded; invasive arterial pressure was measured *via* the radial artery; and cardiac output was measured by the PRAM (Mostcare^®^, Vygon, Italy) method; finally, central venous saturation was monitored.

The hypnotics used in every patient included sevoflurane before surgery and propofol immediately after surgery and until extubation. Doses were adjusted according to BIS XP^®^ (Aspect Medical Systems, Newton, MA) values.

### Data Collected

The following data were collected from all patients: age, sex, EuroSCORE II (perioperative risk of mortality for patients after cardiac surgery), extracorporeal circulation (ECC) time, and aortic clamping time.

The following parameters were measured in blood: hematimetry, troponin I, creatine kinase, lactate, NT-proBNP, and S100B protein. These data were collected at diagnosis (preoperative values) and at 24-h intervals during the first 48 h. S100B protein was only measured at diagnosis and at 24 h.

Hemodynamic measurements: cardiac output and systolic volume index were measured using the Mostcare^®^ monitor. Heart rate, mean arterial pressure, and right ventricular end-diastolic pressure were also determined. These measurements were performed at diagnosis and at 12-h intervals during the first 48 h.

Renal function measurements: the AKI stage of patients was assessed during the first 48 h after recruitment.

Ultrasound measurements: systolic and diastolic peak values, and vascular resistance index of the middle cerebral and lobar artery measured at diagnosis and at 24 h after treatment started.

### Statistical Analysis

Normality of distribution of quantitative variables was assessed by the Shapiro-Wilk test. When *p*-value was <0.05, normality was tested with the P-P curve. If values were normally distributed, Levene test was performed to assess homogeneity of variance. When homogeneity was confirmed, mean values were compared using *t-*test. Results were expressed as confidence intervals. The mean values for the quantitative variables that did not present a normal distribution were compared by the Wilcoxon test, and median values were compared by the Bonett-Price method. Differences between qualitative variables were determined by the Newcombe’s method.

All parameters were analyzed using STATA version 16 software (STATA Corporation).

Sample size was calculated from mean creatinine values in our series of patients with a diagnosis of low cardiac output syndrome (1.010 mg/dl +/− 0.375 mg/dl; with intention to detect a difference between means of 0.3 mg/dl (to detect a levosimendan-induced change of 30% in creatinine), which indicated a change in AKI stage. Based on Epidat 4.0 software (Sergas), 26 patients were needed per group to find statistically significant differences, with a power of 80% (1 – *β* = 0.8) at a significance level of 5% (*α* = 0.05). Assuming an expected loss of 15% patients, the required sample size was 30 patients per group.

The analysis of results was designed to perform an intention-to-treat analysis, assuming drop outs, withdrawals, loss to follow-up, or lack of adherence to the protocol.

## Results

No significant differences were found between groups with regard to the epidemiological variables registered. Accordingly, the epidemiological and intraoperative data for each group did not show significant variations (**Table 1**). Weight and height in the dobutamine group were 78 +/− 12 kilograms, 166 +/− 10 centimeters, and 77 +/− 10.66 kilograms, 166 +/− 9 centimeters. (p > 0.05) for the LEVOSIMENDAN group.

**Table 1:** Epidemiological, hemodynamic, biochemical, and ultrasound basal data; and days in ICU and hospital.

	DOBUTAMINE (30 patients)	LEVOSIMENDAN (30 patients)
Weight	78 +/− 12	77 +/− 10.66
Height	166 +/−10	166 +/− 9.34
Age	68 +/− 9	65 +/− 10
Sex (M/F)	20/10	18/12
EuroSCORE II	2.4 +/− 2.18	2.3 +/− 2.09
Type of surgery:		
Aortic valve	7% (2)	17% (4)
Mitral valve	23% (6)	7% (2)
CABG	13% (4)	10% (3)
Aortic artery	10% (3)	7% (2)
Polyvalvular	20% (5)	28% (8)
Coronary valve	27% (8)	31% (9)
Other	0%	7% (2)
ECC	100 +/− 36	111 +/− 43
Ischemia	76 +/− 27	78 +/−37
Preoperative creatinine	0.99 +/− 0.26	1 +/− 0.25
CI	1.96 +/− 0.23	1.89 +/− 0.32
CVS	67.6 +/− 7.79	61.5 +/− 10.71*
SVI	27.6 +/− 7.40	23.2 +/− 5.74*
Troponin	10.18 +/− 7.08	12.40 +/− 21.58
NT-proBNP	1,393 +/− 1,465	4,051 +/− 3,960*
Lactate	2 +/− 0.84	1.94 +/− 0.92
Days in ICU	5 +/−1	4 +/− 1
Days in hospital	11 +/− 4	10 +/− 4

CABG, coronary artery bypass graft; CI, cardiac index; CVS, central venous saturation; ECC, extracorporeal circulation; ICU, intensive care unit; SVI, systolic volume index. *p < 0.05.

Of the parameters measured, NT-proBNP at diagnosis, systolic volume index (SVI), and central venous saturation (6% lower) were significantly lower in the dobutamine group, as compared to the levosimendan group (*p* < 0.05) (**Table 2**).

**Table 2:** Evolution of hemodynamic indicators in the dobutamine group after 24 and 48 h of treatment.

	DOBUTAMINE	LEVOSIMENDAN
LCOS		
DxMAP (mmHg)	72 +/− 12	73 +/− 10
DxSVI (ml/m^2^)	27 +/− 7	23 +/− 5
DxCI (L/min/m^2^)	1.9 +/− 0.2	1.8 +/− 0.3
DxCVS (%)	67.6 +/− 7.7	61.5 +/− 10.6
DxCVP (mmHg)	8 +/− 3	12 +/− 5
Creatinine (mg/dl)	1.08 +/− 0.40	1.3 +/− 0.39*
Renal VRI	0.69 +/− 0.10	0.70 +/− 0.09
Brain VRI	0.68 +/− 0.15	0.72 +/− 0.12
24 HOURS		
MAP (mmHg)	77 +/− 7	77 +/− 9
SVI (ml/m^2^)	31 +/− 6	30 +/− 6
CI (L/min/m^2^)	2.4 +/− 0.3	2.5 +/− 0.4
CVS (%)	70.5 +/− 6.8	69.6 +/− 5
CVP (mmHg)	10 +/− 4	10 +/− 3
Creatinine (mg/dl)	1.17 +/− 0.4	1.19 +/− 0.4
Renal VRI	0.68 +/− 0.13	0.72 +/− 0.12
Brain VRI	0.72 +/− 0.03	0.77 +/− 0.06
48 HOURS		
MAP (mmHg)	79 +/− 11	76 +/− 8
SVI (ml/m^2^)	30 +/− 8	32 +/− 8
CI (L/min/m^2^)	2.5 +/− 0.5	2.5 +/− 0.6
CVS (%)	68.8 +/− 9.1	71.2 +/− 5.89
CVP (mmHg)	10 +/− 4	10 +/− 3
Creatinine (mg/dl)	1.18 +/− 0.4	1.21 +/− 0.5

CI, cardiac index; CVP, central venous pressure; CVS, central venous saturation; Dx, diagnosis; LCOS, low cardiac output syndrome; MAP, mean arterial pressure; SVI, systolic volume index; VRI, vascular resistance index. “*” statistically significant difference.

Although no significant differences were observed between groups in preoperative creatinine levels, there were significant differences in baseline creatinine levels at the time of recruitment, with baseline levels of 1.08 mg/dl +/− 0.4 in the dobutamine group *vs* 1.39 mg/dl +/− 0.39 in the levosimendan group (p < 0.05).

**Figure 1:**
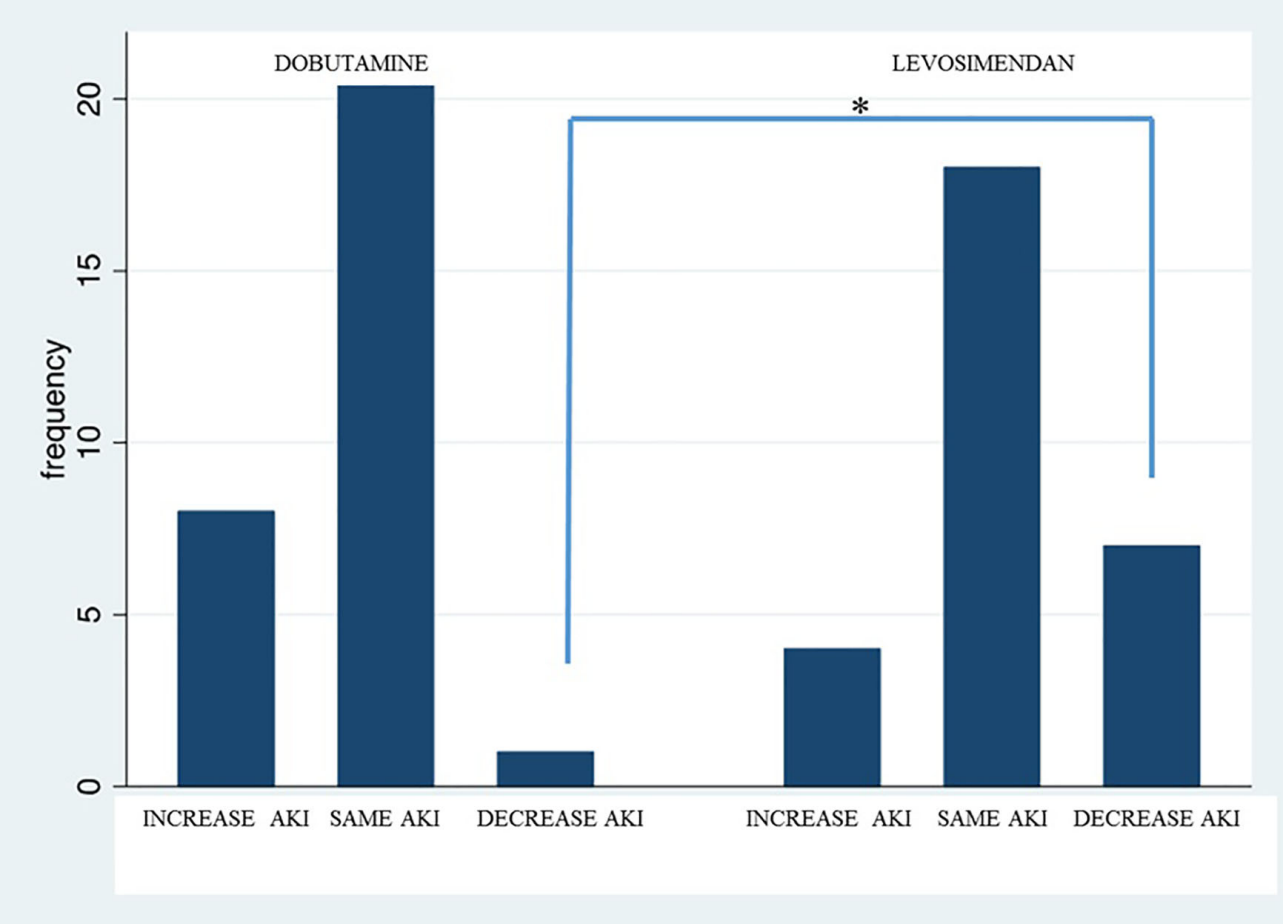
Variation of AKI stage after 24 h in patients treated with dobutamine *vs.* levosimendan. “*” statistically significant difference.

In total, 83% of the patients treated with dobutamine *vs.* 46% of patients in the levosimendan group exhibited an AKI stage of 0 at diagnosis, with a significant difference of 37% between groups. Twenty-four hours after treatment, 28% of patients treated with dobutamine had an advanced AKI stage *vs.* 15% in the levosimendan group, with a non-significant difference of 12% between groups. Additionally, AKI stage improved in 23% of patients treated with levosimendan *vs.* 3% of patients treated with dobutamine, with a significant difference of 20% (p < 0.05) (**Figure 1**).

**Figure 2:**
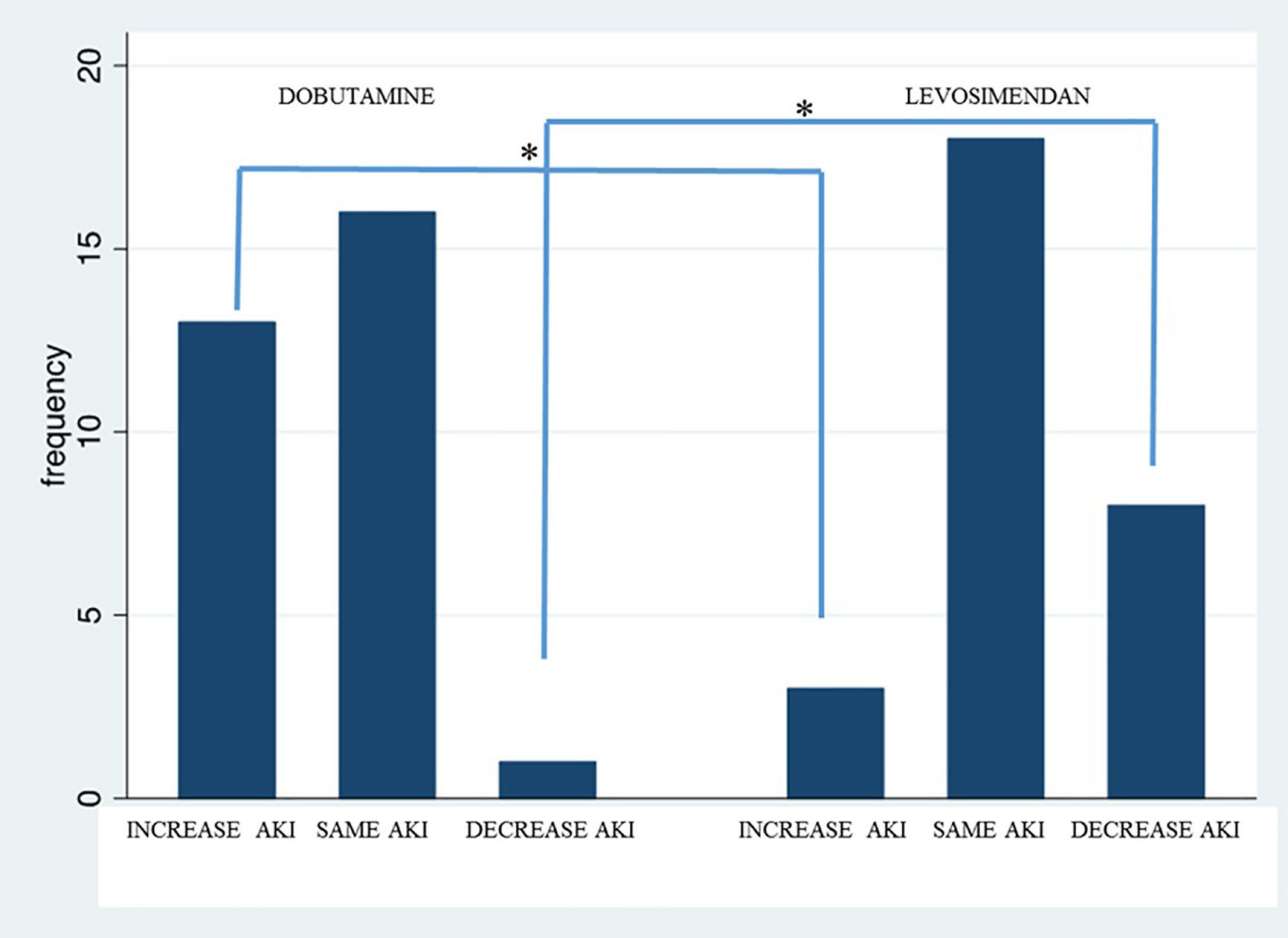
Variation of AKI stage after 48 h in patients treated with dobutamine *vs.* levosimendan. “*” statistically significant difference.

After 48 h, AKI stage worsened in 45% of patients in the dobutamine group *vs.* 12% of patients in the levosimendan group, with a significant difference of 33% (*p* < 0.05). In contrast, AKI stage at 48 h improved in 3% of patients in the dobutamine group and 27% of patients in the levosimendan group, with a significant difference of 24% (*p* < 0.05) (**Figure 2**).

There were no significant differences in hemodynamic parameters neither after 24 h nor after 48 h. Likewise, no significant differences were found between groups in renal VRI values at diagnosis and after 24 h.

Finally, no significant differences were observed in S100B protein levels or in baseline or 24-h VRI values in the middle cerebral artery (*p* > 0.05).

## Discussion

When LCOS is treated with levosimendan, renal function improves at 24 and 48 h of treatment, as compared to dobutamine. The renal protective effects of levosimendan were assessed based on of cardiac function, renal blood flow, and biochemical parameters (creatinine).

In our study, the hemodynamic targets pursued with the treatment of LCOS were reached in both groups. Otherwise said cardiac index (CI) and central venous saturation (SvO_2_) were normalized after the administration of dobutamine or levosimendan.

At the time of recruitment, levels of NT-proBNP were significantly higher in the levosimendan group than in the dobutamine group. This difference shows a poorer baseline cardiovascular status of patients allocated to the levosimendan group. It is worth noting that high NT-proBNP levels have been associated with a higher incidence of renal impairment after cardiac surgery (Patel et al., 2012; Holm et al., 2014; Kato et al., 2017).

Also, there were differences in SVI and SvO_2_ values between groups, with the dobutamine exhibiting higher levels. The decrease in these parameters is associated with a lower oxygen supply to organs (Mezger et al., 2017) and can be explained by a poor baseline cardiovascular status, as reflected by the NT-proBNP levels of the patients who were treated later with levosimendan.

At 24 and 48 h after treatment, no significant differences were observed between groups in hemodynamic or oxygen transport parameters.

At the time of recruitment, renal function parameters showed significant differences between groups, although 1:1 randomization was performed. A higher incidence of renal impairment was observed in the levosimendan group, which caused a significant bias between groups. Thus, increased preoperative levels of creatinine have been shown to be an independent risk factor for acute kidney injury after cardiac surgery. In addition, patients in the levosimendan group had a higher stage of renal insufficiency on AKI scale (Grayson et al., 2003; Stallwood et al., 2004; Kallel et al., 2013; Corredor et al., 2016; Kang and Wu, 2019).

Afterwards, levels of creatinine increased in the dobutamine group and decreased in the levosimendan group, showing significant variations at 24 and 48 h after treatment in favor of the levosimendan group.

There is cumulative evidence that levosimendan improves renal function in patients who have undergone recent cardiac surgery (Bragadottir et al., 2013; Baysal et al., 2014; Guerrero-Orriach et al., 2016; Guerrero Orriach et al., 2017; CHEETAH Study Group, 2018; Qiang et al., 2018; Wang et al., 2019). Nevertheless, the positive effects of the preoperative administration of levosimendan have been recently questioned (Cholley et al., 2017). According to some studies, levosimendan exerts renal protective effects (described elsewhere) by improving cardiovascular function (Silva et al., 2016). However, similar hemodynamic values were observed in the two treatment groups of our study. Therefore, differences in kidney function cannot be explained by cardiovascular or hemodynamic status or oxygen supply/demand balance, since these parameters were similar in the two treatment groups. Potential variations in renal blood flow were examined by renal ultrasonography. Renal function at baseline and at 24 h was evaluated based on variations in VRI, an ultrasound prognostic marker of renal impairment. Decreased VRI values after treatment indicated that organ perfusion had improved as a result of an increased difference between systolic and diastolic flow. In our study, no significant differences were observed between the two treatment groups.

LCOS is established based on different parameters (hemodynamic, clinical, oxygen supply/demand balance) that reflect organ hypoperfusion, which especially damages the kidneys (Pérez Vela et al., 2012; Lomivorotov et al., 2017). When perfusion is restored in a territory affected by ischemia, oxygen deficiency in that tissue may be reversed. However, reperfusion may cause more organic and systemic damage than ischemia. In our opinion, reperfusion during LCOS may serve as a clinical model to assess the protective effects of certain drugs from the deleterious effects of LCOS therapies. Accordingly, the agents used to treat LCOS may have a dual action: inotropic (required to revert LCOS) and pharmacological postconditioning. The mechanism by which several drugs induce pharmacological postconditioning seems to be mediated by the influx of potassium through mitochondrial membrane channels, a therapeutic effect of levosimendan that has been previously described (Du Toit et al., 2008; Buchholz et al., 2014; Stroethoff et al., 2019).

In our study, no significant differences were observed in hemodynamic or ultrasound parameters between groups. Levosimendan and dobutamine exerted differential effects on glomerular filtration rate. Levosimendan prevailingly causes vasodilation of afferent arterioles. In contrast, dobutamine induces a balanced vasodilation of both, afferent and efferent arterioles, thereby increasing renal blood flow, while glomerular filtration pressure is maintained constant. This could explain that renal function improved in the levosimendan group and it is not detected by renal ultrasound. The improvement in renal function is another effect of levosimendan that distinguishes it from beta-agonists. Patients in the group with a poorer baseline cardiovascular status and renal function probably benefited from the postconditioning effect of levosimendan. As shown by their SvO_2_ and NT-proBNP basal values, the levosimendan group had a greater oxygen deficiency than the dobutamine group (Cholley et al., 2017).

In this study, we also evaluated the central nervous system (CNS) using a specific biochemical marker of cell damage (S100B protein). We decided to use the S100B protein in our study as it is a good marker of neural damage or ischemia, since it is released by the glial cells of the brain (Silva et al., 2016). An ultrasonography of the middle cerebral artery was performed to measure parameters of brain vascular perfusion which indicate variations in systolic and diastolic arterial flow. No significant differences were found between the two groups in VRI values, and neurological effects directly depend on cerebral blood flow.

No significant differences were found in S100B protein levels between groups. However, there were baseline differences, with a higher incidence of renal and cardiac impairment in the levosimendan group as compared to the dobutamine group. Renal and cardiac dysfunction indicates a higher risk for postoperative neurological dysfunction (Pack 2 score). On the other hand, increased levels of NT-proBNP have been associated with a higher risk of neurological damage.

The lack of significant differences between groups in S100B levels can be explained by several factors. First of all, levosimendan may exert a greater postconditioning effect on the CNS than on the kidneys. However, its effect may have been underestimated in the levosimendan group, which had a greater oxygen deficit, as they had a higher risk during organ reperfusion. The fact that neural damage parameters did not deteriorate in any group might reflect a higher neurological preservation in the levosimendan group. On the other hand, most patients of the two groups received mediastinal blood, which could have increased S100B protein levels. However, S100B levels were homogeneously distributed in both groups, since mediastinal blood reinfusion is a common technique in our institution. Moreover, neurological effects depend directly on cerebral blood flow, but differences were not observed between groups; therefore, this effect cannot be explained by cerebral blood flow (Silva et al., 2016).

This study has some limitations and strengths. First of all, this is a single-center study. Secondly, neurological examination was not performed using a clinical test (i.e. gold standard evaluation) because many patients were under the effects of several drugs for pain treatment, mainly opioids. We thought that an early clinical assessment of neurological damage might be influenced by these effects, and thus differences in the biochemical markers of brain damage would be more objective. However, the use of the S100B protein in this group of patients could have also been biased by mediastinal blood reinfusion, a standardized technique in our institution. Our study suggests a possible mechanism by which calcium sensitizers may exert beneficial effects on renal and brain function. Biochemical, ultrasound, and cardiocirculatory parameters indicate an improvement of renal function. The stability of SB 100 proteins may reflect an effect based on postconditioning.

## Conclusion

The renal function of patients with LCOS after cardiac surgery only improved in patients treated with levosimendan as compared to dobutamine. Neurologically, no significant differences were observed between groups in S100B protein levels, although, initially, patients in the levosimendan group had a higher risk for neurological dysfunction.

## Data Availability Statement

All datasets generated for this study are included in the article/supplementary material.

## Ethics Statement

The studies involving human participants were reviewed and approved by CEI MALAGA NORTE. The patients/participants provided their written informed consent to participate in this study.

## Author Contributions

JG-O, AM-M, MRA: Conception and design. AF, MG-O, IM-C, AR-F, DA-V, JE-B, GQ-M, ES-H, SR-M: Acquisition of data. IB-E, AG-L, MR-N: Revising it critically for important intellectual content. JA-T, CS-F: Acquisition of data, analysis and interpretation of data. LG-S, JC-M: Conception and interpretation of data. All authors contributed to the article and approved the submitted version.

## Funding

LG is supported by the Miguel Servet program from the ISCIII (Spain) (“Miguel Servet II” program, CPII18/00030) and Nicolas Monardes program from the Consejería de Salud de Andalucía (Spain) (C-0028-2018). CS is supported by a grant from the ISCIII (Spain) (“PFIS” program, FI16/00241). CIBER Fisiopatología de la Obesidad y Nutrición (CIBEROBN) is an ISCIII project (Spain).

## Conflict of Interest

The authors declare that the research was conducted in the absence of any commercial or financial relationships that could be construed as a potential conflict of interest.

The handling editor declared a past co-authorship with one of the authors JG.

## Abbreviations

FIM-BGC, FIMABIS-LOW CARDIAC OUTPUT; AKI scale, Acute kidney injury scale; CI, Cardiac index; CNS, Central nervous system; SvO_2_, Central venous saturation; EuroSCORE II, perioperative risk of mortality for patients after cardiac surgery; ECC, Extracorporeal circulation; LCOS, Low cardiac output syndrome; SVI, Systolic volume index; VRI, Vascular resistances index.
